# Microbe Profile: Durusdinium trenchii: a thermotolerant coral symbiont

**DOI:** 10.1099/mic.0.001679

**Published:** 2026-05-07

**Authors:** Kiran-Kumar Shivaiah, Sabrina L. Rosset, Robert A. Quinn

**Affiliations:** 1 Department of Biochemistry and Molecular Biology, Michigan State University, East Lansing, MI, USA; 2 Hawaiʻi Institute of Marine Biology, University of Hawaiʻi at Mānoa, Kāneʻohe bay, HI, 96744, USA; 3 Department of Microbiology, Genetics and Immunology, Michigan State University, East Lansing, MI, USA

**Keywords:** Corals, coral reefs, *Durusdinium*, Symbiodiniaceae, symbiont

## Abstract

*Durusdinium trenchii* is a unicellular dinoflagellate in the family Symbiodiniaceae, a diverse group of photosynthetic microalgae known for forming symbiotic relationships with cnidarians and other reef organisms. Notably, this species displays exceptional tolerance to heat stress, enabling it to persist within the coral gastrodermis and often dominate symbiont communities under elevated temperatures. *D. trenchii* can confer increased thermal tolerance and reduced bleaching susceptibility to corals, though frequently with trade-offs in host growth and calcification. Its resilience has been linked to genome duplication, photoprotective mechanisms and characteristic lipid profiles. Its unique molecular traits, host generalist nature, ecological flexibility and increasing prevalence in warming oceans underscore the importance of this microbe in coral reef responses to climate change.

## Graphical Abstract

*Durusdinium trenchii* – a heat-tolerant algal endosymbiont of reef corals.

(a) At ambient sea surface temperatures (SSTs), *D. trenchii* typically occurs at low background levels, especially in regions where this symbiont is non-native (e.g. parts of the Caribbean).

(b) Prolonged thermal stress caused by climate-change-induced rising SSTs can trigger symbiont shuffling, in which *D. trenchii* and other heat-tolerant algae become dominant within the symbiont communities of certain coral species or individuals, reducing their bleaching susceptibility. This shift can carry trade-offs, such as a reduced coral growth rate; corals that lack *D. trenchii* or fail to shuffle bleach.

(c) When SSTs return to ambient levels, the prevalence of *D. trenchii* often declines back to background levels due to energetic trade-offs, and native, heat-sensitive symbionts regain dominance.

*Created with BioRender.com

## Taxonomy

Phylum: Dinoflagellata; class: Dinophyceae; order: Suessiales; family: Symbiodiniaceae; genus: *Durusdinium*; species: *trenchii.*

## Properties

*Durusdinium trenchii* is a unicellular dinoflagellate belonging to the family Symbiodiniaceae. Morphologically, *D. trenchii* cells are typically spherical to oval and measure ∼9.8–10.4 µm in diameter, placing them at the mid-range for the Symbiodiniaceae [1]. While a few Symbiodiniaceae species are facultative symbionts – capable of living both freely in the water column and in association with host animals – many others exist primarily in symbiosis. Symbiodiniaceae form symbiotic associations with hosts such as cnidarians (including corals, jellyfish and sea anemones), molluscs, sponges and other coral reef organisms. *D. trenchii* is largely host-associated, forming stable and long-term symbioses with cnidarians, though it is not entirely obligate.

The cnidaria–dinoflagellate symbiosis plays a pivotal role in tropical marine ecosystems, forming the primary biological unit of a reef, the scleractinian coral. *D. trenchii* resides within the coral’s gastrodermis, the innermost tissue layer that borders the gastrovascular cavity, encased in host-derived membranes known as symbiosomes. Through photosynthesis, these symbiotic algae uniquely supply the coral host with essential organic carbon and other nutrients, a function critical to coral survival in nutrient-poor environments. This mutualistic relationship is fundamental to the health and productivity of coral reefs, but its stability is under threat, particularly due to anthropogenic climate change. At prolonged elevated temperatures, corals lose their symbionts in a phenomenon known as coral bleaching. If these symbionts are not re-established after the thermal stress subsides, the coral becomes deprived of essential nutrients, leading to starvation, increased susceptibility to disease and ultimately death. It has been widely observed that corals hosting *D. trenchii* are more resistant to heat-induced bleaching. *Durusdinium*-hosting corals are thereby more tolerant to elevated temperatures and temperature fluctuations than those hosting many other dinoflagellates [2]. These traits make it a particularly interesting and ecologically important member of the Symbiodiniaceae that will likely play a prominent role in the future of tropical reefs.

## Genome

The total assembled length of the genome of *D. trenchii* is ∼670.43 Mb, with a G+C content of 47.4 mol%. The number of protein-encoding genes is reported to be 30,054, with an average gene length of 15.03 Kbp [3]. Several studies have suggested that whole-genome duplications have occurred in the genus *Durusdinium* [1]. *D. trenchii* is characterized by multiple diallelic microsatellite loci; other species of the genus often exhibit similar patterns. These diallelic patterns support the hypothesis of genome duplication during the evolution of this species [1]. Furthermore, one study, based on genome assembly analysis, predicted that this whole-genome duplication may bolster the heat tolerance of *D. trenchii* [3] by allowing for increased gene expression and functional redundancy, thereby providing a buffer against thermal stress [3]. Importantly, this duplication appears to have been primarily selected during the free-living life phase, and interestingly, subsequent divergence of duplicated genes may have enabled novel functions that further support thermal tolerance during symbiosis [3].

## Phylogeny

*D. trenchii* is a species of dinoflagellate symbiont within the family Symbiodiniaceae [1] . Initially classified under the genus *Symbiodinium*, as *Symbiodinium trenchii*, this organism was reclassified into the genus *Durusdinium* following taxonomic revisions in 2018 [1]. Before this reclassification, it was commonly identified by its Internal Transcribed Spacer 2 (ITS2) sub-cladal nomenclature as ITS2 type D1a, which was part of the former clade-based system (Clade D) used for Symbiodiniaceae. The reclassification was based on genetic and physiological distinctions among the Symbiodinaceae that warranted a major phylogenetic overhaul. Molecular phylogenetics suggests that *D. trenchii* has a close evolutionary relationship with other *Durusdinium* species but remains distinct from members of the more common *Cladocopium* genus, which typically dominates symbioses in stable reef environments across much of the Indo-Pacific, though patterns vary regionally [4,5].

## Key features and discoveries

Geographically, *D. trenchii* is distributed primarily across the Indo-Pacific region, but it has expanded into the Caribbean Sea, though at lower genetic diversity [4]. Its presence is notably prominent in areas subjected to elevated sea temperatures, where it often becomes the dominant symbiont in corals experiencing thermal stress. The thermal resilience of *D. trenchii* has been a focal point of research, especially in the context of global climate change and its impact on coral reef ecosystems [4].

During thermal stress events, corals have been observed to replace or change the relative abundances of their resident/native symbiotic algae, favouring the heat-tolerant, host generalist *Durusdinium* [5]. A host-generalist is a symbiont capable of associating with a wide range of coral species rather than being restricted to a single host lineage, a trait that enables *D. trenchii* to rapidly colonize diverse corals following bleaching events. This dynamic adjustment, often referred to as symbiont shuffling, refers to the modification of the proportions of existing symbiont types within the coral host rather than acquiring entirely new symbionts. By increasing the dominance of more thermally tolerant species already present in the symbiont community, corals can better withstand elevated temperatures without forming a completely new symbiosis. Studies have shown that even trace amounts of *D. trenchii* can enhance the bleaching response of corals, delaying the onset of bleaching under heat stress conditions. It is also worth noting, however, that these shuffling events do not always occur, and a few studies have demonstrated that, over longer time periods, corals will reshuffle again back to the dominance of their native symbionts [5]. The recent increased prevalence of *D. trenchii* in the Caribbean is believed to be due to repeated ocean heat waves in the region and subsequent coral bleaching events [4].

Several studies have investigated the mechanisms underlying this heat tolerance [6]. So far, few conclusions have been drawn on the exact molecular processes behind the resilient nature of *Durusdinium*, but evidence is mounting. Corals hosting *D. trenchii* exhibit upregulation of genes associated with heat stress response [7]. Conversely, a symbiosis with *Durusdinium* induces a downregulation of host genes related to extracellular structures and the metabolism of carbohydrates and lipids. Surprisingly, the gene expression pattern induced by *D. trenchii* in control (non-stressed) conditions resembles that of *Cladocopium*-hosting corals under heat stress. This implies that *D. trenchii* preconditions corals for thermal stress, possibly enhancing their baseline heat tolerance. This is very similar to the phenomenon called ‘frontloading’, which is characterized by a higher baseline expression of certain genes, particularly those associated with heat stress responses [7].

It has also been suggested that the lipid composition of cellular membranes plays an important role in the resistance of *Durusdinium* to higher temperatures, particularly relating to thylakoid integrity [6]. Lipidomic analysis of *D. trenchii* and *Cladocopium C3* isolated from different colonies of *Acropora valida* demonstrated that *D. trenchii* had significantly higher saturation of sulfoquinovosyl diacylglycerols and phosphatidylglycerols, two lipid classes with important roles in photosystem stability. *D. trenchii* was also characterized by a higher ratio of di- to mono-galactosyldiacylglycerols (DGDG:MGDG) [6]. An increased ratio of DGDG:MGDG, the principal membrane constituents of thylakoids, is a known biochemical mechanism of thermal tolerance in plants. Thermal stress consequently resulted in extensive thylakoid membrane remodelling by the *Cladocopium* strain, but not by *D. trenchii*, supporting the proposition that *D. trenchii*’s thermal tolerance is mediated by greater inherent thylakoid stability. The same study also highlighted that *D. trenchii* is distinguished from *Cladocopium* by its higher abundance of monoacyl forms (lysolipids) of its major non-plastidial membrane lipids, 1,2-diacylglyceryl-3-(o-carboxyhydroxymethylcholine) and phosphatidylcholine [6]. However, the mechanism by which lysolipid synthesis relates to bleaching resistance remains unknown. Investigations into the photophysiological responses of *D. trenchii* under heat stress have demonstrated its capacity to maintain photosynthetic efficiency at elevated temperatures. While some studies suggest this microorganism exhibits similar thermal tolerance to other Symbiodiniaceae species up to certain temperatures (25–30 °C), its ability to sustain photosynthetic activity under prolonged heat stress is likely a key factor contributing to the delayed or reduced bleaching response of corals associated with *D. trenchii* [8].

While the heat tolerance conferred by *D. trenchii* is beneficial, there may be ecological trade-offs for its host. Some studies have suggested that the energetic demands of maintaining a symbiosis with *D. trenchii* could impact coral growth, reproduction and overall fitness [9]. Studies have shown that corals hosting *D. trenchii* exhibit slower rates of calcification compared with those hosting *Cladocopium*, and *D. trenchii* has been shown to retain more of the fixed carbon for its own metabolism instead of translocating it to the host – a ‘selfish’ strategy that prioritizes symbiont survival over host growth [9]. These apparent trade-offs, however, are influenced by environmental conditions and specific host–symbiont interactions. For example, limitations in coral growth rates are largely eliminated under warmer conditions (1.5–3 °C above ambient), when growth rates of corals harbouring heat-sensitive symbionts of the genus *Cladocopium* decline significantly [9]. Furthermore, these trade-offs may be minimized or absent when *D. trenchii* is a native symbiont, as co-evolved partnerships can maintain higher nutrient exchange and growth performance even under heat stress [10]. Taken together, these findings suggest that the costs and benefits of hosting heat-tolerant symbionts are context-dependent and shaped by evolutionary history, emphasizing the need to consider both environmental variability and symbiont and coral host identity when predicting coral resilience.

## Summary

*D. trenchii* stands out as a vital symbiotic partner for corals, especially under conditions of thermal stress. Its heat-tolerance mechanisms, including gene expression modulation, membrane biochemistry, whole-genome duplication and photophysiological adaptations, contribute significantly to the resilience of coral reef ecosystems. However, this resilience must be understood within the broader ecological context of coral–algal symbioses. Host specificity plays a critical role: while host-generalists like *D. trenchii* can temporarily dominate under stress, corals often reacquire their native symbionts once conditions normalize [5]. Moreover, the invasive spread of *D. trenchii* in the Caribbean and potential trade-offs, such as reduced calcification and loss of genetic diversity, highlights that thermotolerance may not always be favoured over the long term [4]. In a future of even warmer oceans and increased bleaching events, one of the most important questions in coral reef ecology and microbiology is: will thermally tolerant species and strains of symbiotic dinoflagellates, such as *D. trenchii*, be naturally selected by corals worldwide? While *D. trenchii* can confer short-term thermal benefits, its long-term ecological dynamics – including host-specificity, competitive interactions and potential trade-offs – remain unclear. Future research should focus on how this naturally thermotolerant symbiont contributes to coral survival and the persistence of diverse Symbiodiniaceae lineages, rather than assuming universal positive selection.

## Open questions/major questions

Will *D. trenchii* spread worldwide as corals experience more thermal stress in warming oceans?

How stable and persistent is *D. trenchii* in corals once environmental conditions return to baseline?

Which duplicated genes and regulatory changes underlie the adaptive traits of *D. trenchii?*

How do different strains of *D. trenchii* vary in their thermal tolerance and metabolic performance, and what environmental factors drive this variation?

What physiological trade-offs do corals experience when hosting *D. trenchii*, particularly in terms of growth, calcification and overall fitness under non-stressful conditions?

## Abbreviations

DGDG:MGDG: Digalactosyldiacylglycerols
ITS2: Internal Transcribed Spacer 2
MGDG: Monogalactosyldiacylglycerols
